# The development of patient-specific 3D anatomical models in minimally invasive parathyroidectomy

**DOI:** 10.3389/fendo.2024.1514451

**Published:** 2024-12-11

**Authors:** Zahra J. Haq, Ahmed Ahmed, Alaa Abdelsalam, Soudeh Chegini, Tom R. Kurzawinski, Simon Morley, Mark McGurk, Tarek Abdel-Aziz

**Affiliations:** ^1^ University College London Medical School, University College London, London, United Kingdom; ^2^ Department of Endocrine Surgery, University College London Hospital, London, United Kingdom; ^3^ Oral and Maxillofacial Surgery, University College London Hospital, London, United Kingdom; ^4^ Department of Imaging, University College London Hospital, London, United Kingdom; ^5^ Oral, Maxillofacial, Head and Neck Surgery University College London Hospital, London, United Kingdom

**Keywords:** primary hyper parathyroidism, parathyroid - adenoma, parathyrodectomy, minimally invasive surgery, HoloLens 2, three-dimensional mode

## Abstract

**Background:**

Surgery is the preferred treatment for primary hyperparathyroidism. Minimally invasive parathyroidectomy is only feasible with accurate preoperative localisation. Virtual 3D anatomical models can be constructed from patient-specific CT scans using segmentation software.

**Methods:**

We aimed to create virtual 3D models from 4D-CT scans of parathyroid tumours using segmentation technology. We designed a small pilot study to assess the utility of 3D models within surgical practice. We assessed surgeon, trainee and patients’ opinion and satisfaction with the models. The NASA TLX survey was the primary data collection tool

**Results:**

Creation of novel 3D models was achieved, these featured a 360-degree axis of rotation and transparency mode to assist in surgical planning. Models were used intraoperatively with the HoloLens 2 headset to locate parathyroid tumours real time before surgery. Total mean workloads for surgery planning when averaged revealed a decrease workload (39.45 vs 27.45) points with adjunctive use of models (p=0.002). Mental demand showed the greatest decrease in mean workload out of all the 6 subscales tested for in the NASA TLX (210.3 vs 136.7) points. Patient satisfaction score was statistically significant for the difference before and after seeing the 3D model regarding anatomical location (p=≤0.001),

**Conclusion:**

In this work, we developed patient-specific virtual 3D anatomical models of parathyroid tumours for use in surgery using novel techniques, previously never applied to parathyroidectomy. Our initial success in model construction and subsequent opinion of surgeons, trainees and patients contributes to the developing body of literature in favour of virtual modelling for parathyroidectomy.

## 1 Introduction

Primary hyperparathyroidism (PHPT) has a prevalence of approximately 233 per 100,000 women in the United States, notably higher in black and white women aged 70-79 years (1–3). Minimally invasive parathyroidectomy (MIP) is the preferred surgical approach for single gland disease, especially when coupled with intraoperative parathyroid hormone monitoring (IOPTH) (4) Our group has also shown that a newer platform of ultrafast IOPTH is accurate, simple and quicker (5).

Compared to bilateral neck exploration, MIP offers a reduced operation time, a smaller incision, and reduced tissue dissection (6, 7). However, MIP is dependent on the surgeon’s ability to visualise and plan the operation based on pre-operative imaging. There is a natural variance in visualisation power and experience of surgeons- with aphantasia, the absence of visualisation ability, being an extreme example (affecting up to 5% of the global population), with more people than originally thought suffering from partial aphantasia (8).

Primary operative difficulties centralise around ectopic and multi-gland disease with ectopic glands making up to 16% of cases – such cases often require more extensive imaging (9). Poor outcomes are particularly associated with revision surgery with re-explorative complications occurring in 27% of patients (10, 11).

Current preoperative imaging for parathyroid localisation relies on ultrasound, sestamibi and/or 4D CT replacing sestamibi as the primary diagnostic tool (12–14). Drawbacks centralise around the 2D nature of images represented on a monitor. A high cognitive demand is placed on surgeons, with planning reliant on their ability to visualise the patient 3D anatomy from these slices and reports.

Patient-specific virtual 3D anatomical modelling has proven to be a rapidly expanding area within surgery showing extensive potential in surgical planning, training and patient education. This novel form of preoperative imaging has become commonplace is various surgical specialties namely hepatobiliary and urological surgery and has yet to be introduced to endocrine surgery (15, 16). Virtual neck exploration for parathyroid surgery has a reported sensitivity greater than US sestamibi and CT imaging whilst also reducing the risk of post-operative hypoparathyroidism due to greater localisation ability (17).

The first aim of this work was to explore the feasibility of creating 3D models for parathyroid tumours. We also assessed the feasibility and applicability of converting these 3D models into holograms and uploading them to an ergonomic holographic device (Microsoft HoloLens 2). The hologram would then be superimposed onto the patient’s neck blending elements of the real world and digital world, in what is known as mixed reality.

The second aim was to objectively measure surgeon workloads and collected surgeon and trainee opinions regarding the models with a questionnaire and feedback forms to measure the efficacy in surgical planning, patient education and surgical training.

The third aim was to compare the patient baseline understanding of parathyroid anatomy and surgery during routine consenting and after showing them the 3D reconstructed model.

## 2 Methodology

### 2.1 Model construction

3D parathyroid models were created based on the reconstruction of the 4D CT scans of the parathyroid tumours using the 3D slicer 5.6.2 version (18). Each structure in the neck has a different radio density measured quantitatively by Hounsfield units (HU).

This depends on reconstruction of the thyroid gland and parathyroid adenoma with the identification of the relations between them which would help the surgeon predict the exact localization of the adenoma.

In order to superimpose the 3D models over the patient’s neck we needed to transform the 3D models into 3D holograms. These holograms would then be uploaded to the HoloLens2 headset where they could be visualised as 3D holograms and superimposed over the patient after being anaesthetised.

A transparency mode would be applied when required to the models to help with the identification of parathyroid tumours that were located deep to the thyroid gland.

Cases were randomly selected from an MDT at our unit. Each case was modelled using preoperative CT images, models included; surrounding vertebrae and bone, trachea, cricoid cartilage, thyroid and parathyroid adenoma tissue.

All digital imaging and communications for medical purposes (DICOM) images of patient parathyroid CT scans were anonymised and processed externally, these were all arterial phase CT scans. All 3D models were constructed by an external company (www.medicalar.co.uk) with two medical physics technicians who regularly produce 3D models (19).

See **Appendix I** for further methodology.

### 2.2 Utility of models in practice

We designed a small pilot study at our centre to assess the feasibility and utility of models within surgical practice. We divided this into 3 stages: surgeon and trainee opinion of the models, initial benefits of using models in the preoperative setting and patient satisfaction regarding the models.

#### 2.2.1 Preoperative planning

Six surgeons were asked to assess 3D parathyroid models of five patients with a diagnosis of PHPT and who had a CT scan for localisation of the parathyroid tumour. Cases were randomly selected from a multidisciplinary team (MDT) meeting at our unit.

**Figure 1:**
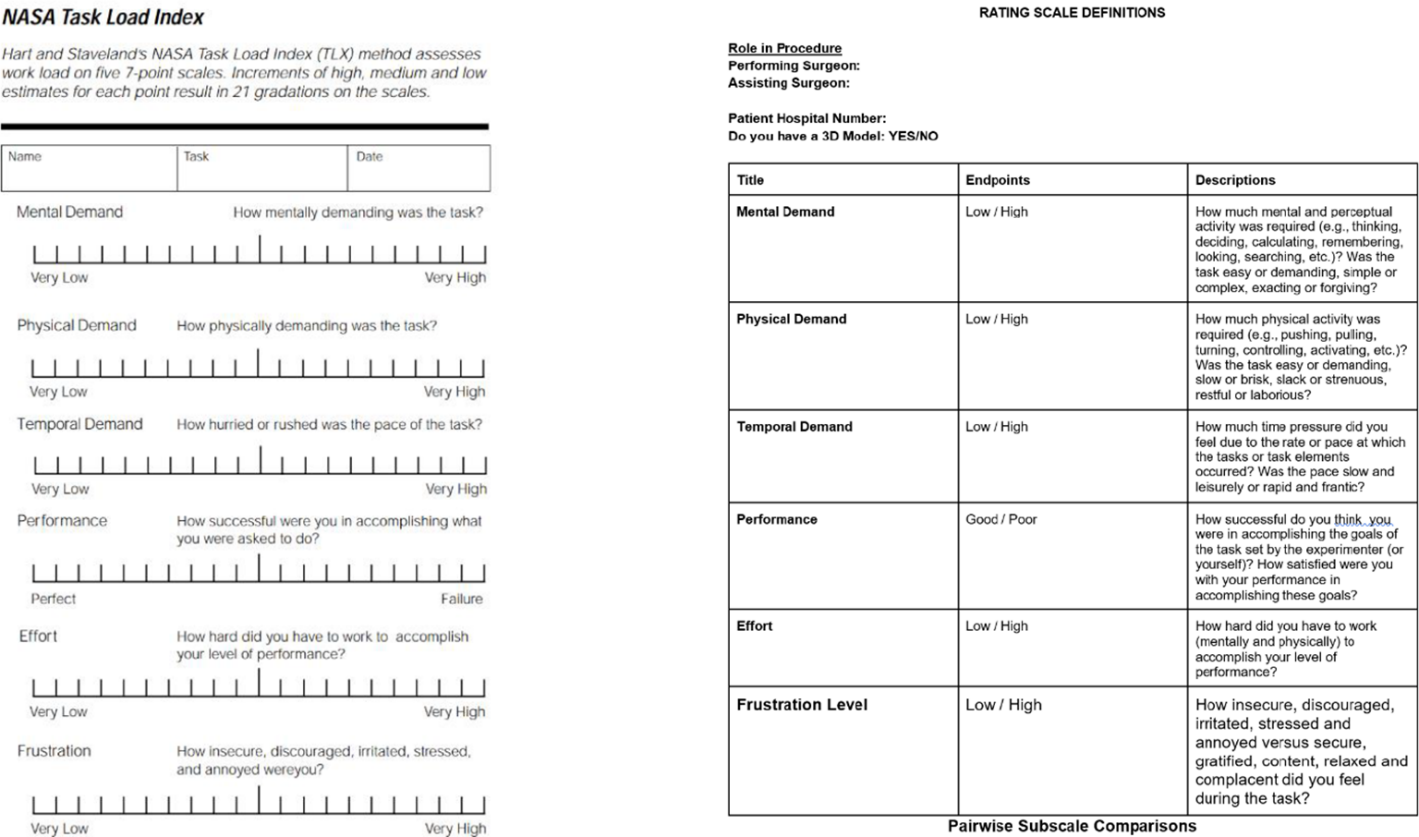
NASA task load index form (20).

The NASA Task Load Index (TLX) survey was the primary data collection tool (20). The NASA TLX form features six subscales: mental demand, physical demand, performance effort and frustration, with surgeons rating each category from 1-20 based on perceived task difficulty (**Figure 1**). The NASA TLX is the most common assessment tool for perceived task workload retrospective to task accomplishment (21). The surgeons’ task required them to access a specific patient’s record, review relevant medical records and plan the hypothetical parathyroidectomy procedure based off this. They first completed the NASA TLX form labelled ‘no model’ and then the form labelled ‘model’ for a different patient within the cohort of selected patients, now having access to the 3D model in addition to patient records. Protocol was repeated for each of the patient cases per surgeon. There was no time restriction applied.

The surgeons were then required to respond to 11 statement-based questions evaluating their thoughts on the models as a new resource, statements were scored from; 1 being strongly disagree to 10 being strongly agree.

#### 2.2.2 Trainee feedback

Three senior trainee surgeons were asked to give their opinions on the models through a questionnaire. Trainees viewed a prototype virtual 3D model and subsequently filled out a questionnaire establishing the value of models in surgical training for MIP surgery. The responses were documented as a descriptive evaluation.

#### 2.2.3 Patient satisfaction

A separate group of 9 patients with PHPT were prospectively randomly selected from a separate MDT.

Surgical technique and planning was discussed with the patients, the corresponding 3D parathyroid model was then shown and described in relation to possible complications. Patient understanding and satisfaction was assessed with a questionnaire, repeated before and after seeing the 3D model

### 2.3 Statistical analysis

Weightings and subscale scores from NASA TLX forms were entered into the official NASA TLX Application giving a continuous value between 1-100 (low-high) (22–24). Surgeon workload scores for each case were averaged to give the mean workload per patient. The values for planning with and without model were compared. Statistical evaluation of workload means was carried out via a one-tailed paired student t-test following a Shapiro-Wilk normality test. These p values were compared to a 0.05 significance level. All statistical calculation was performed on Excel version 2019 (25).

## 3 Results

### 3.1 The reconstruction of the 3D models

Successful generation of virtual 3D anatomical models was achieved accomplishing the primary aim. It was possible to create accurate 3D models of the parathyroid tumours through segmentation of the 4D CT scans and soft tissue reconstruction.

**Figure 2:**
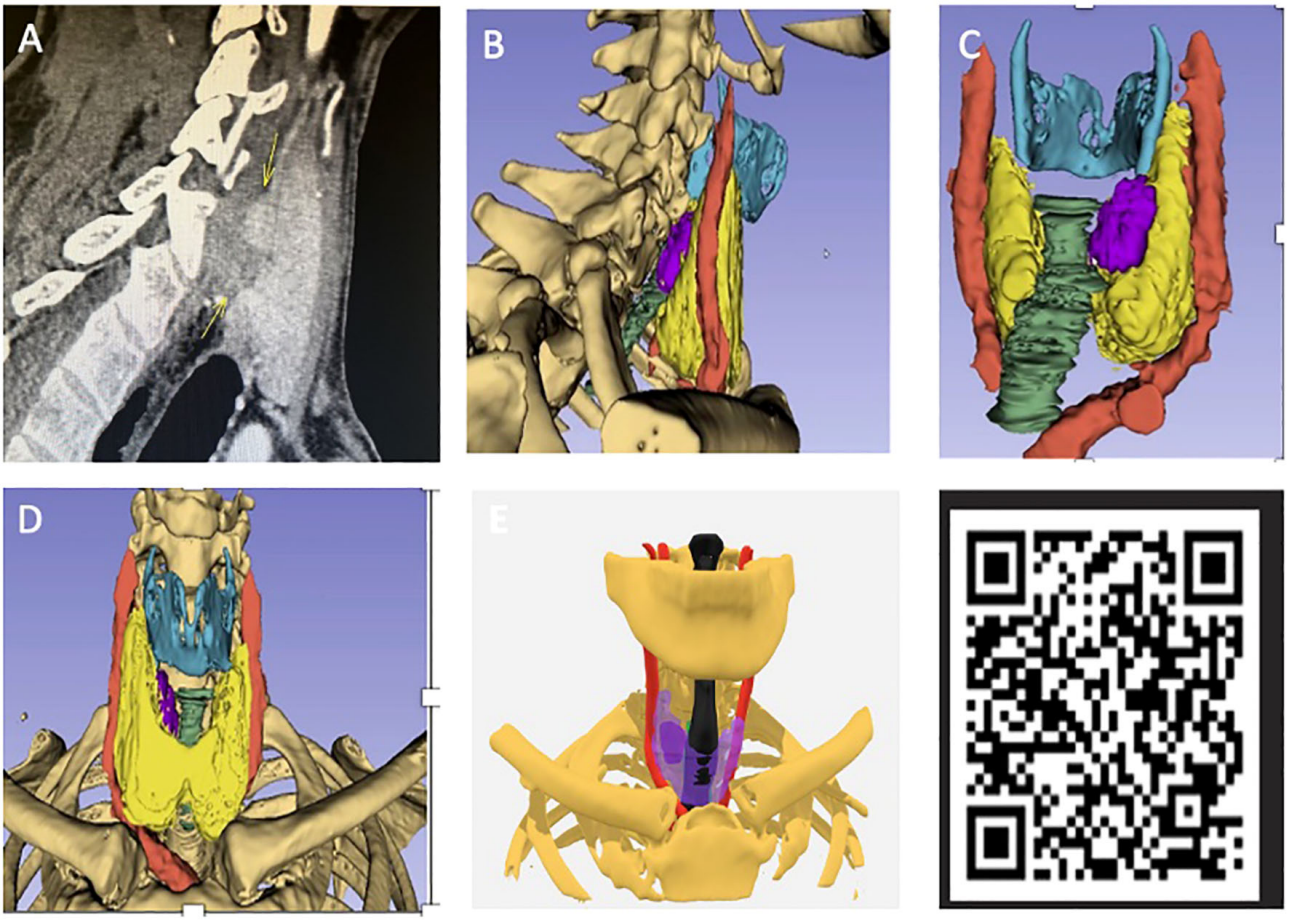
**(A)** Preoperative scan for patient 2 from pilot study with position of adenoma labelled (see **Table 1**). **(B)** Virtual 3D model sagittal view. **(C)** Model from posterior angle with adenoma labelled (purple) and bone made transparent. **(D)** Front view with no transparency. **(E)** Adjustable transparency of anatomical structures allows for direct visualisation of parathyroid glands through the thyroid gland which has been made semi-transparent (purple). QR code which can be scanned via smart phone to view model on hand-held device.

The 3D models were compatible with mobile phone/interactive devices after anonymising patient data and could be manipulated by the user featuring a 360-degree axis of rotation as well as an interactive interface. A transparency mode was also included to visualise the parathyroid tumours by looking through the thyroid gland which would suit posteriorly located tumours (**Figure 2**).

### 3.2 The creation of the holograms and superimposition

The second aim was to create 3D holograms and to superimpose these holograms over the patient’s neck in the operating room to assist with real-time localisation and accurate placement of the incision for MIP. In our experience, the location of the parathyroid tumours would variably change after positioning the patient in neck extension after being anaesthetised as opposed to the neutral positioning during 4D CT scans, therefore we decided to also reconstruct fixed bony structures which could be relied on as fixed anatomical landmarks. These structures would not be affected by neck movement or extension during surgery. Both clavicles and specifically the sternoclavicular joints were reconstructed. The mentum was also reconstructed as a midline bony landmark to assist in the triangulation method which is discussed below.

**Figure 3:**
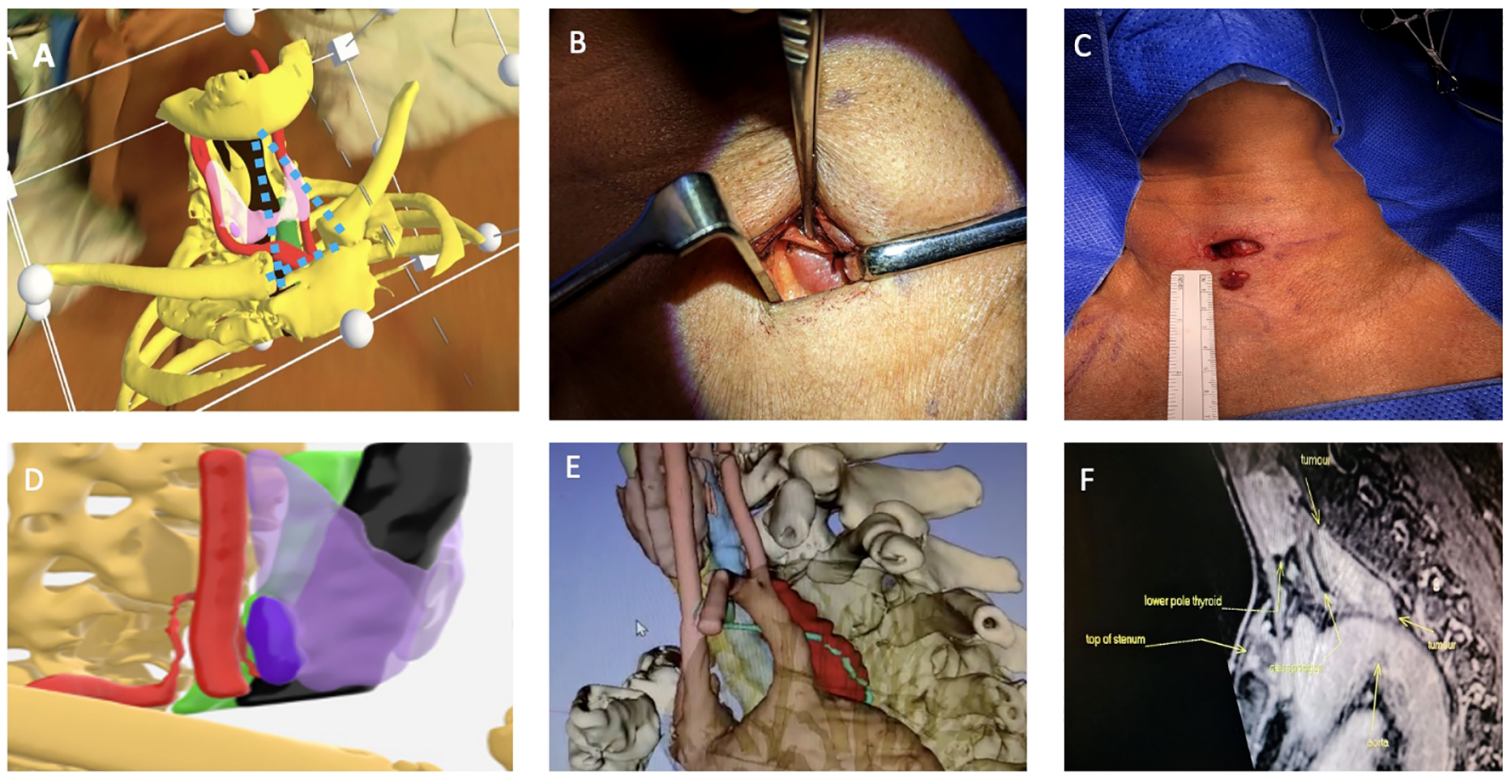
Top row **(A)** Surgeon’s view from the right side of the operating table of 3D model superimposition on patient’s neck using Microsoft HoloLens2,the parathyroid adenoma (Right lower pink structure), thyroid (light purple in transparency), carotid artery (red) and bones (yellow), imaginary triangulation dotted lines in blue. **(B)** Shows incision placement and immediate location of the parathyroid adenoma with minimal dissection and no subplatysmal flap elevation. **(C)** Postoperative excision with maintained wound length as there was no need for excessive retraction. Bottom row **(D)** It shows side-view of the 3D model where ITA and its branch which supplies the parathyroid adenoma are reconstructed, in this model, thyroid gland (light purple), right parathyroid adenoma (deep purple), trachea (black) and carotid artery (red). **(E)** shows a very large parathyroid tumour in the prevertebral space closely related to the aortic arch and the thoracic duct (lime green). **(F)** MRI sagittal view shows relation of parathyroid adenoma to aortic arch but thoracic duct was more difficult to visualise. The thoracic duct was identified and ligated at the start of the dissection to avoid a thoracic duct injury and a chyle leak.

To allow for accurate superimposition, we developed a novel triangulation technique which involved constructing an imaginary triangle through the HoloLens 2 between the virtual mentum and sternoclavicular joints which would be superimposed with precision over the patient’s bony structures. We would then mark the exact site of the parathyroid tumour by looking at patient’s neck from the front and the side through the HoloLens in the supine position prior to neck extension. This helped to enhance the accuracy of virtual 3D models when used via the HoloLens 2 virtual reality headset during neck extension (**Figure 3**).

### 3.3 Refinement of the 3D models

Throughout construction methods to overcome previously never encountered hurdles were developed. The most challenging and potentially serious anatomical point during minimal invasive parathyroidectomies is the relation between the parathyroid adenoma and the recurrent laryngeal nerve (RLN) especially when dissecting superior parathyroid adenomas. The RLN cannot be identified on CT scans and consequently couldn’t be reconstructed in 3D models. The RLN is known to be closely related to the inferior thyroid artery (ITA) which led us to try to reconstruct and map out the ITA and its branch to the parathyroid adenoma (**Figure 3D**).

Also, with advancements in this technology and 3D models, we were also able to utilise MRI scans and reconstruct parathyroid tumours and related structures. This is particularly important in giant parathyroid tumours due to the intricacy of the surrounding structures. (**Figures 3E, F**).

### 3.4 Model utility and 3D model characteristics

Results were divided into three stages; surgeon and trainee feedback on the utility of models in practice, a pilot study into the potential benefits of using models to plan surgery and patient opinions of the models. The basis of which to collate surgeon opinions and whether sufficient and plausible uses for the models in practice is apparent.

Patient age range was 33-65years (median: 59, mean: 54 years), with 1 male and 4 females (**Table 1**). Localised abnormal parathyroid glands were predominantly single (4) with the majority being left inferior (3), other locations include right sided and left superior – with one bilateral re-operative case (patient 4). There were no reported operative complications and no evidence of disease recurrence at 6 months follow up.

**Table 1:** Patient demographics tumour characteristics and operative notes.

Patient	Age (years)	Sex	Location of Adenoma	Size of Adenoma cm	Co-morbidities	Nature of Surgery	Highest IOPTH pg/mL	15 min IOPTH pg/mL	Percentage IOPTH reduction
**1**	65	F	Left sided adenoma posterior to lower lobe of the thyroid	2.2x0.9x0.5	Hypertension Ankylosing Spondylitis	MIP	52.7	10.2	80.6
**2**	55	M	Right sided parathyroid adenoma	1.2x1.5x2.4	–	MIP	126.3	17.6	86.0
**3**	60	F	Posterior to lower left lobe of thyroid	0.7x0.5x1.6	Hypothyroidism	MIP	37.8	6.1	83.8
**4**	33	F	Left inferior parathyroid glands	–	Recurrent primary hyperparathyroidism	Bilateral neck exploration: Left lower, right superior and right inferior glands excised (reoperative procedure, previous left hemithyroidectomy).	82.2	14.8	81.9
**5**	59	F	Left superior parathyroid adenoma	1.4x0.4x0.4	–	MIP	56.7	6.0	89.4

### 3.5 Surgeon feedback

Statement based questions showed high agreement throughout, with statements 4, 3 and 1 averaging the highest. The scoring system ranges from 1 (strongly disagree) - 10 (strongly agree) with 8/11 statements averaging over a 9 in score. A surgeons questionnaire (**Appendix-1**) showed that 10/11 statements correspond to a p value <0.05 establishing a large majority of surgeon opinion in favour of the models. An extremely high level of agreement is seen for 45% of statements (p <0.001).

### 3.6 Surgical trainee feedback

Three surgical trainees (two fellows and one ST5 registrar) from the practice with an average of 11 years’ experience were asked to review a prototype model for training and visualisation purposes. Trainees critiqued the prototype with respect to its utility for their training. They were required to respond to a pre- prepared questionnaire. This feedback can be summarised as a descriptive evaluation:

*Current challenges of MIP training:*

The most common challenge identified was difficulties finding the adenoma on imaging and in practice. Trainees expressed that it was challenging to visualise complex 3D anatomical relations relative to the adenoma.

*Benefits of using the model for training:*

After viewing the prototype model all trainees agreed that it would be useful to their training. Key benefits included improved ability to localise the adenoma due to greater insight into anatomical location and increased depth perception.

*Challenges to training overcome by model complement:*

Trainees expressed that models would enhance the training experience for challenging cases, allowing them to visualise and plan their approach. All trainees agreed that using the models for training would ‘increase (their) confidence when operating’ particularly in ‘placing the incision’.

*Improvements to design and comments:*

Trainees agreed the addition of the RLN would be helpful. This currently cannot be added due to the absence of nerves on the CT scans used to create the models. It was also not possible to visualise the RLN on the MRI scans performed in this study.

Mean Weighted Workload Comparison Overall, a significantly reduced perceived workload after the additional use of the model with 4/5 (80%) of the cases having a p value <0.05 in all cases except patient case 3 where the additional use of the model did not make a significant improvement to the average surgeon-perceived workload of planning.

The most statistically significant case was patient 4 (p<0.001) with a mean workload of 53.89 (no model) compared with 32.33 for the model group. Patient case 3 was not found to be statistically significant with p >0.05 (0.077) and means of 39.95 and 31.45, indicating a minimal additive benefit with the model.

The mean represents on average, a ‘medium’ workload for no model usage compared to a ‘low’ workload for use of the model. The supplemental use of 3D models could thus lessen the cognitive demands of MIP planning on the operating surgeon.

Workloads for patient 4 show the largest difference (21.56) followed by patient 1 (12.34) and 5 (12.00) with 2 (8.50) having the smallest difference in mean workloads.

**Figure 4:**
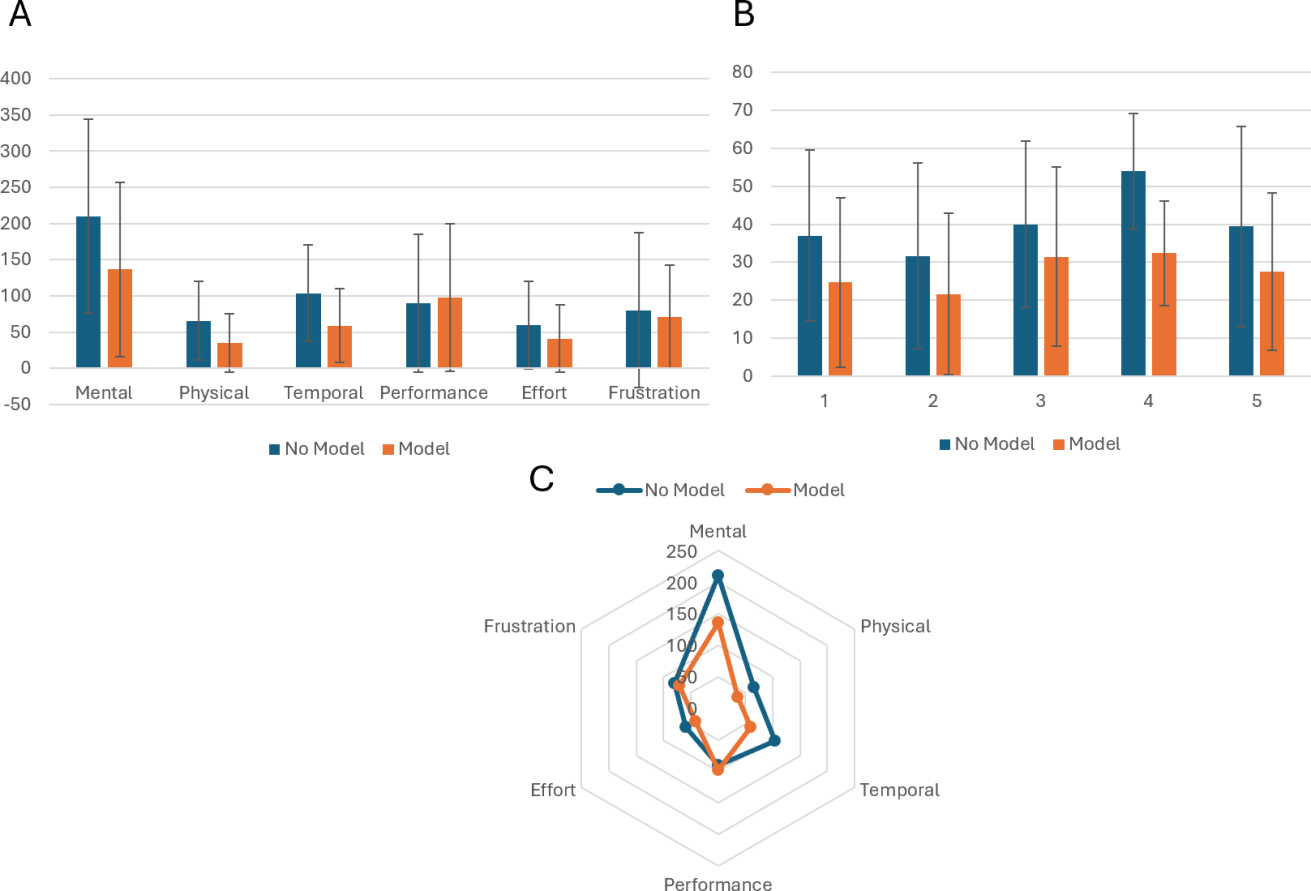
**(A)** Bar chart comparing workload scores for individual cases with and without model adjunct. **(B)** Panel **A** Bar chart showing the mean weighted values for each of the 6 subscales on the NASA TLX. **(C)** Radar chart showing greatest decrease in mental workload with temporal demand showing the second greatest decrease.

Mental demand showed the greatest decrease in mean workload out of all the 6 subscales tested for in the NASA TLX (210.3 vs 136.7). Mental demand was collectively ranked highest in weighted importance by the 5 surgeons participating (**Figure 4**). Temporal demand has the second largest decrease (44.83 points) followed by effort (18.17) in favour of the models.

### 3.7 Patient satisfaction

Patient satisfaction score was statistically significant for the difference before and after seeing the 3D model regarding anatomical location (p=≤0.001), relation to important structures (p=≤0.001), possible complications (p=0.004), overall expectations (p=0.007) and wound satisfaction (p=0.02) as seen in **Table 2**.

**Table 2:** Comparison of patient understanding and satisfaction before and after model according to questionnaire.

Questionnaire	Before scan (n=9)	After scan (n=9)	Test of Sig.	p
Anatomical relation				
Mean ± SD.	6.4 ± 1.7	9.7 ± 0.5	t=6.183*	<0.001*
Median (Min.-Max.)	7 (4 - 10)	10 (9 - 10)		
Relation to important structures				
Mean ± SD.	5.7 ± 2.1	9.7 ± 0.5	t=6.000*	<0.001*
Median (Min.-Max.)	6 (2-8)	10 (9 - 10)		
Possible complications				
Mean ± SD.	6.1 ± 2.4	9.3 ± 0.7	t=3.965*	0.004*
Median (Min.-Max.)	6 (2 - 10)	9 (8 - 10)		
Wound satisfaction				
Mean ± SD.	7.9 ± 1.4	9.2 ± 0.8	t=2.828*	0.022*
Median (Min.-Max.)	8 (5-9)	9 (8 - 10)		
Expectations				
Mean ± SD.	6.2 ± 1.3	9.7 ± 0.7	Z=2.692*	0.007*
Median (Min.-Max.)	7 (4 - 8)	10 (8 - 10)		

SD, Standard deviation.

t, Paired t-test.

Z, Wilcoxon signed ranks test.

p, p value for comparing between Before scan and After scan.

*: Statistically significant at p ≤ 0.05.

## 4 Discussion

This project explores the development of virtual 3D parathyroid models for parathyroidectomy surgery. Through the utilisation of segmentation software such as 3D slicer we have shown that 3D modelling is feasible in clinical practice with uses that span from surgical planning and training to enhancing patient consenting. The development of virtual 3D models in this project represents the initial success of segmentation software and a new era of adjunctive imaging in parathyroidectomy surgery.

The novel models described feature 3D planes and 360-degree axis of rotation allowing a surgeon to inspect the parathyroid adenoma from all angles, giving the ability of guiding the incision directly above the adenoma. In challenging cases it would offer a 3D representation of anatomical landmarks which may not be in their expected positions due to fibrosis and previous damage. These interactive features combined with an accurate representation of patient specific bony and soft tissue anatomy has never been amalgamated into a virtual model before.

The addition of the ITA to models is interesting and with further research might allow the path of the RLN to be estimated which is a crucial anatomical landmark and a great advantage of these virtual 3D anatomical models. With expected improvements in segmentation software the course of the RLN is a crucial component to incorporate in future iterations.

The main advantage of 3D models is enhanced depth perception which in practice, can reported by radiologists but requires imagination by surgeons performing the surgery. 3D models enable surgeons to have better view of the depth and relations of the adenoma. It is possible of course that experienced parathyroid surgeons would be able to translate the 4D CT images which show an obvious parathyroid adenoma into a mental model that would map onto the patient’s neck, and many of them would be able to use intraoperative US to assist in this localisation. It is however evident that the newer generations of parathyroid surgeons do not perform US scans routinely and definitely not intraoperatively and find it more difficult to create that mental model which requires years of experience.

Jagadeesan Jayender et al. had previously reported on five patients (11) where he used an intra-operative MRI and a navigating electromagnetic tool for proper localisation of parathyroid adenoma. In Jayender’s study, he created 3D patient-specific models of the skin, trachea, carotid artery, thyroid, and parathyroid adenoma using 3D Slicer. He depended on a modified Bovie pencil that acted as an electromagnetic sensor to assess the position of each structure in three-dimensional space. In Jayender’s study, he reported that all his patients were cured. The main advantage in our technique, we reconstructed 4D CT scans which were done before the surgery so we saved more time than doing an intra-operative scan. The HoloLens2 enabled us to apply 3D models directly on the patients without need for any further electromagnetic probes.

The initial results from our practice reiterate the utility of virtual reality in the planning of parathyroidectomy surgery and the compatibility of our models in this process.

Virtual 3D models can therefore be used throughout the operation- being superimposed on the patient with the aid of the triangulation technique to ensure anatomical landmarks represented on the device align with the patient.

The models were well received by the surgeons involved, commenting that they would devise similar ‘imaginary’ models mentally before every surgery (26). Surgeon confidence in the models is a key factor for their clinical feasibility reinforced through the statement questionnaire administered at the end of the survey. Due to the extremely high scores for 10/11 statements, it can be gathered that there is scope for models to play a prominent role in practice. Moreover, it has been extensively demonstrated that surgeon confidence plays an integral role in operative success (27).

The use of 3D models albeit in its infancy for MIP has shown promise in our centre especially in its initial impact on the workload of planning. In a small pilot study conducted at our centre a decrease in average workload was found (39.45 compared with 27.45, p=0.002) with 80% of the patient cases showing significantly reduced scores (p <0.05). Increased cognitive workloads have previously been related to inferior performance in surgery as well as greater likelihood of errors (28). Our initial impression from data collected at our centre supports the future development and further study of virtual 3D parathyroid models especially for surgical planning offering potential reassurance for complex cases and decreasing mental demands of MIP. Moreover, there is an increasing need to reduce adverse events related to medical errors, which have exhaustively been shown to have detrimental impacts on surgeons and patients alike (29) (30).

Surgical training is also a central area which virtual 3D models has excelled in our study. Simulations implemented at Stanford medical school and Newcastle university use custom anatomical models to replicate clinical cases and complex operations (31). Trainees can therefore familiarise themselves with challenging procedures without endangering the patient, reducing both training costs as well as training hours (32, 33). The Eyesi surgical simulator, endorsed by the Royal College of Ophthalmologists, has been recently integrated into routine ophthalmological training with studies attesting to its ability to improve technical skills required in cataract surgery (34). Positive sentiment towards the use of 3D models was reinforced by the surgical trainees recruited for this study. All trainees reported that use of models would be beneficial for training, potentially increasing their confidence in theatre particularly with placing incisions. It was also mentioned that identification of the RLN would have been helpful, however this was not possible in the current study. Potential future work may focus on incorporating MRI-based data to assist with RLN identification and reconstruction. Moreover, virtual 3D models serve as a valuable tool for patient communication. Incorporating models in the consenting process aligns with legal and ethical foundations of healthcare, reducing patient anxiety and increasing their understanding (35).

This study offers a reproducible method to be applied to different centres with variations in volume and expertise to assist with the standardisation of surgical training. Virtual 3D models may not significantly change the outcomes for experienced surgeons however, this work underpins the key early steps in utilising augmented reality technology with the limitations segmentation software expected to evolve rapidly in the near future.

### 4.1 Limitations

The limitations of 3D virtual parathyroid models are heavily associated with that of the CT scans used to create them. The same difficulties faced during localisation of parathyroid adenomas on CT scans are those that affect 3D representation with smaller adenomas being more challenging to identify. Similarly model consistency is highly dependent on the individual analysing the CT scans and rendering them on 3D Slicer software. This leaves a margin for potential model variation due to the number of different sources of model production. Errors such as these fall into a similar category as that of operator dependency in ultrasound and conventional imaging modalities. The semi-automatic shading of 3D Slicer and other software currently used to make virtual anatomical models requires the practical element of supervision with rendering being a manual process. Each model takes an average of 60 minutes to complete costing approximately £300 however this is a transferrable skill and the 3D slicer software used in this study is a free, open source software. Future studies exploring use of these models for clinically complex and re-operative cases may prove to be a more fruitful avenue possibly showing greater efficiency and significance than blanket manufacture. It would also be beneficial to include a control group in future studies to compare surgical outcomes and to show the impact of the 3D models and the use of the HoloLens 2 on surgical training and performance.

## 5 Conclusion

In conclusion this project has added a novel insight into the development of virtual 3D anatomical models for parathyroidectomy. Our initial success in model construction and subsequent opinion of surgeons and trainees at our centre lends to the growing sentiment towards incorporating virtual 3D modelling to parathyroid surgery and planning. Future applications as well as manifold areas for further study have been brought to light, reiterating the need for technology of this nature in improving the efficacy of surgical practice.

## Data Availability

The original contributions presented in the study are included in the article/**Supplementary Material**. Further inquiries can be directed to the corresponding author.
