# Supplementary material for: The development of patient-specific 3D anatomical models in minimally invasive parathyroidectomy
Source: Front Endocrinol (Lausanne). 2024 Dec 11;15:1514451. doi: 10.3389/fendo.2024.1514451 (PMC11668577; doi:10.3389/fendo.2024.1514451)

Appendix I

**Preoperative Planning**

Six surgeons were asked to assess five patient cases (Table 1). Cases were randomly selected from an MDT at our unit (February 2022). Each case was modelled using preoperative CT images, models included; surrounding vertebrae and bone, trachea, cricoid cartilage, thyroid and parathyroid adenoma tissue. Image segmentation is not commonly taught to clinicians therefore most segmentation was outsourced to specially trained medical physics technicians at our centre with oversight from the radiology department. DICOM CT arterial phase images were uploaded to 3D Slicer software where they were rendered using the software platform. Models constructed therefore represent anatomy present on CT scans used in their construction.

NASA Task Load Index (TLX) forms were the primary data collection resource with two per surgeon labelled ‘model’ and ‘no model’ (Fig. 1 and Fig. 2) [19]. The NASA TLX form contained six subscales; mental demand, physical demand, performance effort and frustration. Surgeons scored each category from 1-20 (low-high) in relation to how they perceived the task (Fig. 3). The NASA Task Load Index is the most common assessment of perceived workload when carrying out a task administered retrospectively to accomplishment [20]. It has been previously used to assess surgeon workload as well as being adapted to form SURG TLX [21]. Surgeons were asked to access a specific patient’s record and review relevant medical records to plan the parathyroidectomy procedure. Surgeons then completed the first NASA TLX form labelled ‘no model’. They then completed the second NASA TLX form labelled ‘model’ this time having access to the model in addition to patient records (Fig. 4 and Fig. 5). Protocol was repeated for each of the patient cases per surgeon. There was no time restriction applied. The surgeons were then required to respond to 11 statement-based questions evaluating their thoughts on the models as a new resource, statements were scored from; 1 being strongly disagree to 10 being **
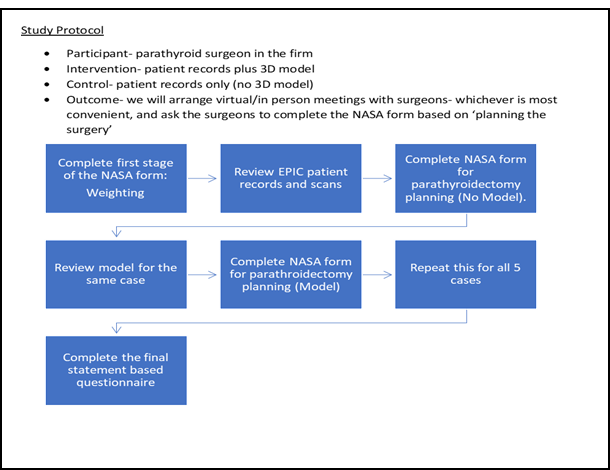
**strongly agree.

**Patient Satisfaction**

A separate group of 9 patients were prospectively randomly selected from a separate MDT (March 2022).

Surgical technique and planning was discussed with the patients with a demonstration on their neck, the 3D parathyroid model was then shown and described in relation to important anatomical structures. Patient understanding and satisfaction was assessed with a questionnaire, using a 0-10 Likert scale, before and after seeing the 3D model (Fig. 6). The aim being to compare baseline patient understanding after the routine consenting discussion to patient understanding after the discussion with the addition of the model.


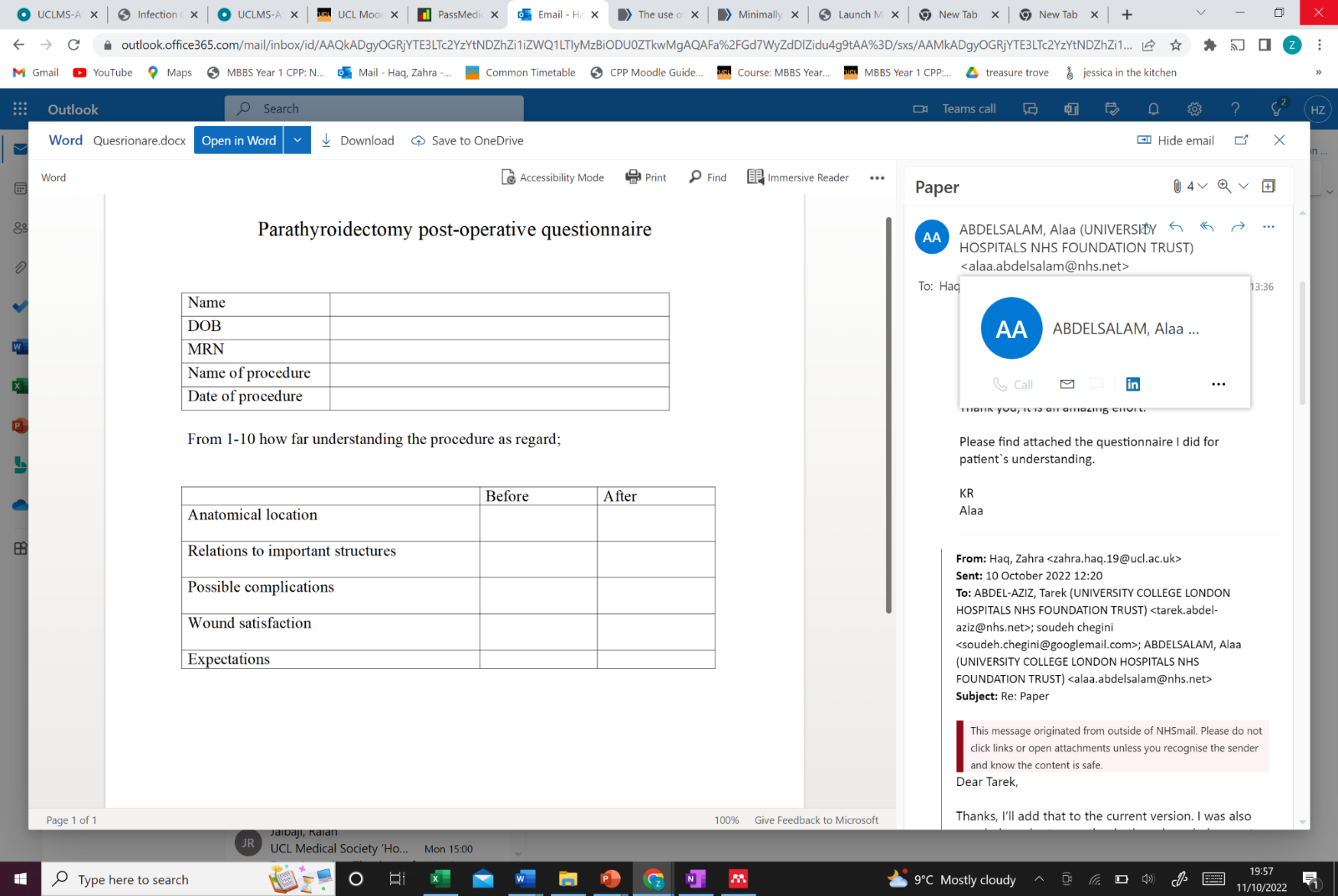


**Surgeon questionnaire and results**


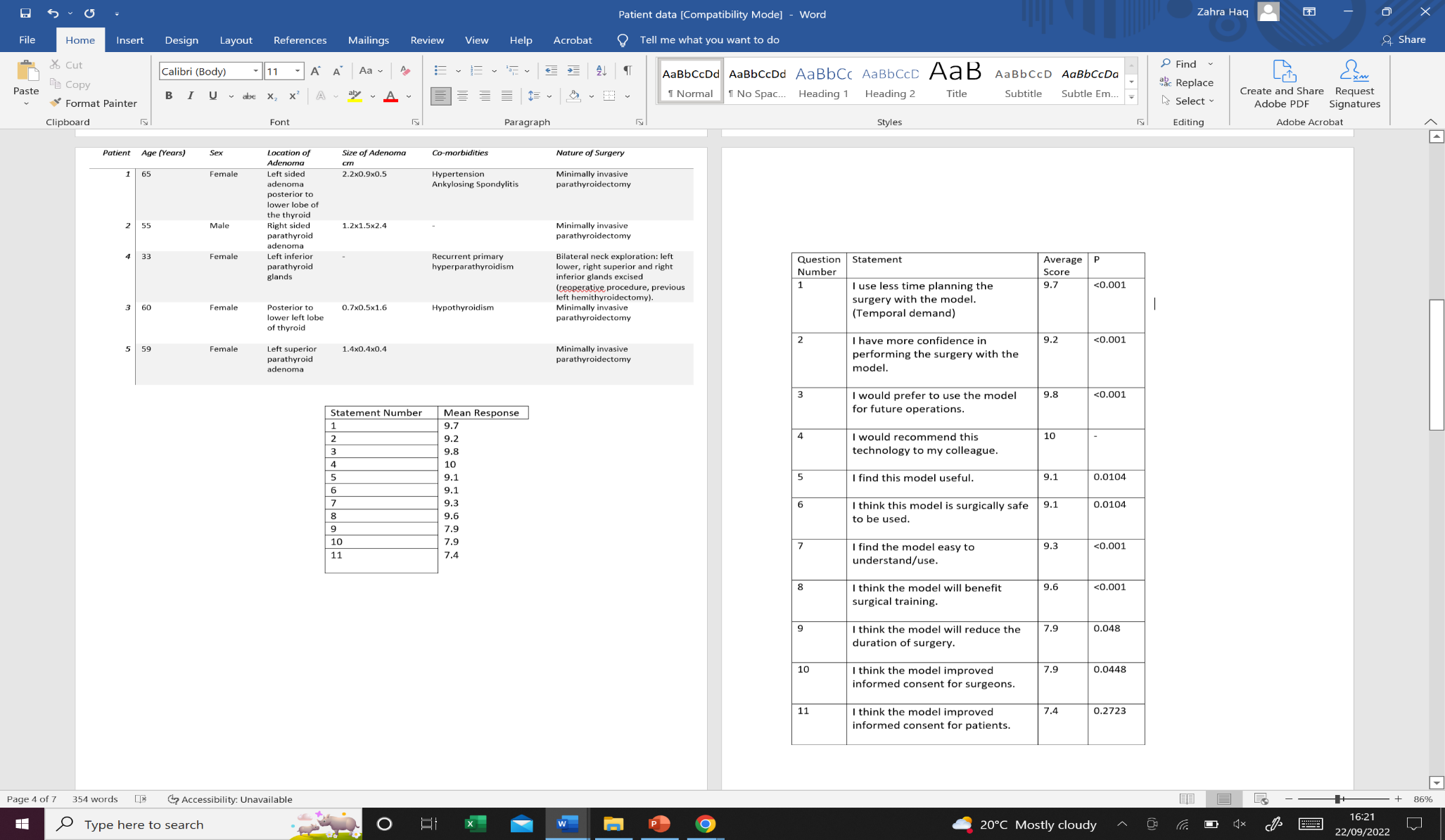

Supplement: Supplementary file 1 [file DataSheet1.docx]
